# Supplementary figures and images for: Intravital imaging-based genetic screen reveals the transcriptional network governing Candida albicans filamentation during mammalian infection
Source: eLife. 2023 Feb 27;12:e85114. doi: 10.7554/eLife.85114 (PMC9995110; doi:10.7554/eLife.85114)

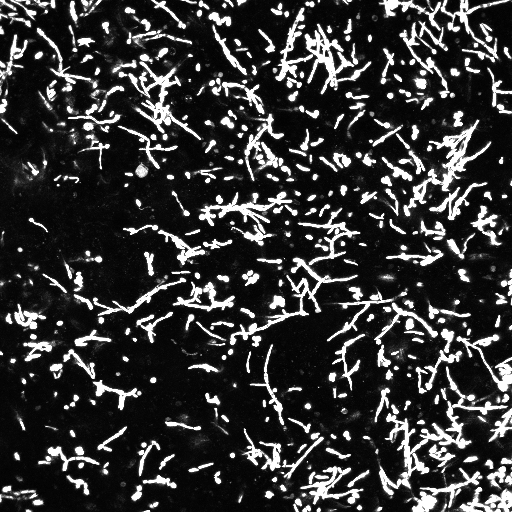

Supplement: Figure 1—source data 1. — Confocal image files for transcription factor mutants with altered filament/yeast distribution along with their wild type (WT) comparator. Files are named: genename_Max Stack_1 for the mutant images and WT_genename_Max Stack 1 for the WT counterpart. [file elife-85114-fig1-data1.zip › Figure 1. A-source data 1/isw2_Max Stack_1.tif]

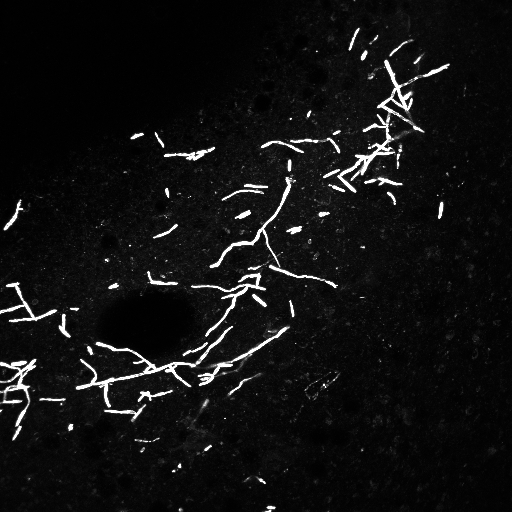

Supplement: Figure 1—source data 1. — Confocal image files for transcription factor mutants with altered filament/yeast distribution along with their wild type (WT) comparator. Files are named: genename_Max Stack_1 for the mutant images and WT_genename_Max Stack 1 for the WT counterpart. [file elife-85114-fig1-data1.zip › Figure 1. A-source data 1/tup1_Max Stack_1.tif]

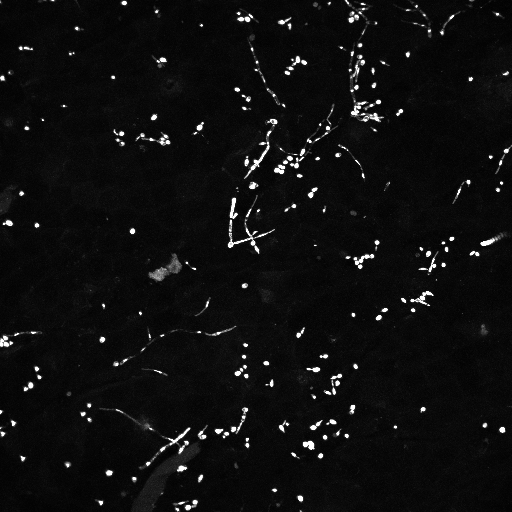

Supplement: Figure 1—source data 1. — Confocal image files for transcription factor mutants with altered filament/yeast distribution along with their wild type (WT) comparator. Files are named: genename_Max Stack_1 for the mutant images and WT_genename_Max Stack 1 for the WT counterpart. [file elife-85114-fig1-data1.zip › Figure 1. A-source data 1/WT_isw2_Max Stack.tif]

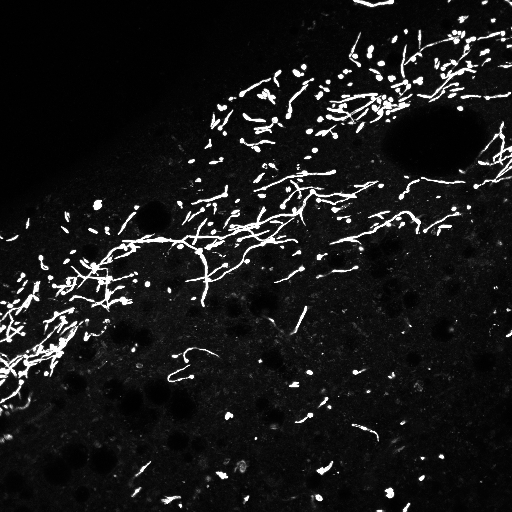

Supplement: Figure 1—source data 1. — Confocal image files for transcription factor mutants with altered filament/yeast distribution along with their wild type (WT) comparator. Files are named: genename_Max Stack_1 for the mutant images and WT_genename_Max Stack 1 for the WT counterpart. [file elife-85114-fig1-data1.zip › Figure 1. A-source data 1/WT_tup1_Max Stack.tif]

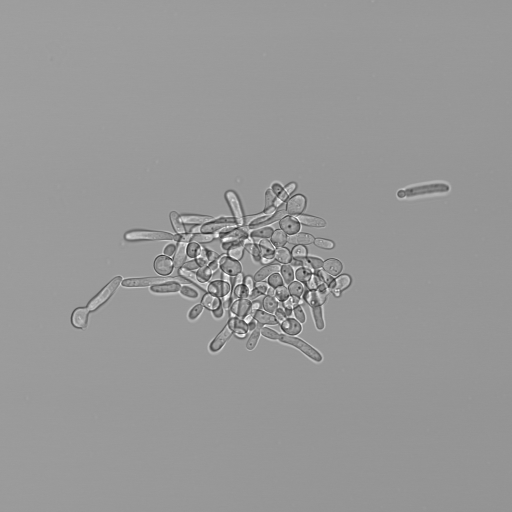

Supplement: Figure 1—source data 3. [file elife-85114-fig1-data3.zip › Figure 1. D-source data 1/brg1_RPMI+10%Serum_4hr.tif]

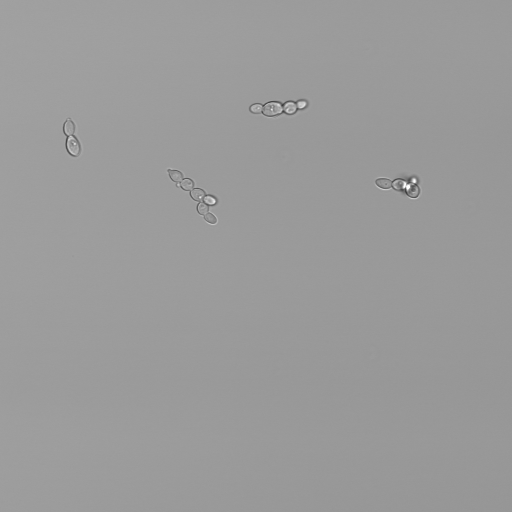

Supplement: Figure 1—source data 3. [file elife-85114-fig1-data3.zip › Figure 1. D-source data 1/rim101_RPMI+10%Serum_4hr.tif]

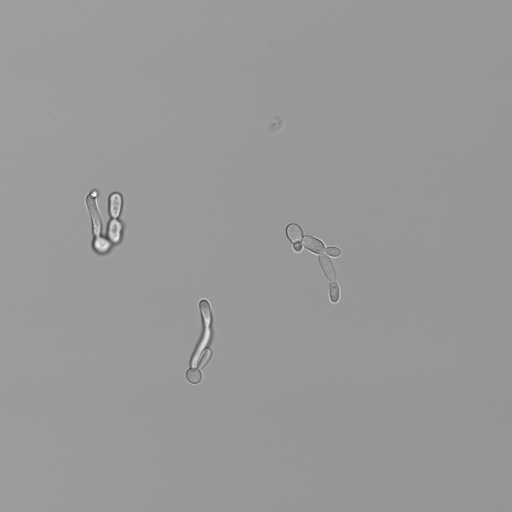

Supplement: Figure 1—source data 3. [file elife-85114-fig1-data3.zip › Figure 1. D-source data 1/rob1_RPMI+10%Serum_4hr.tif]

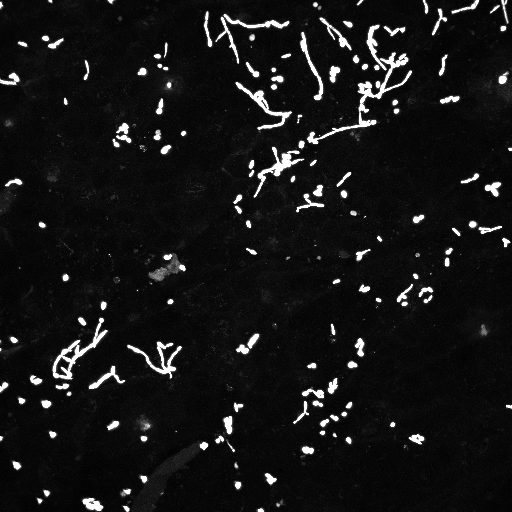

Supplement: Figure 2—source data 1. — Files are named: genename_Max Stack_1 for the mutant images and WT_genename_Max Stack 1 for the WT counterpart. Figure 2A. [file elife-85114-fig2-data1.zip › Figure 2. A-source data 1/isw2_Max Stack.tif]

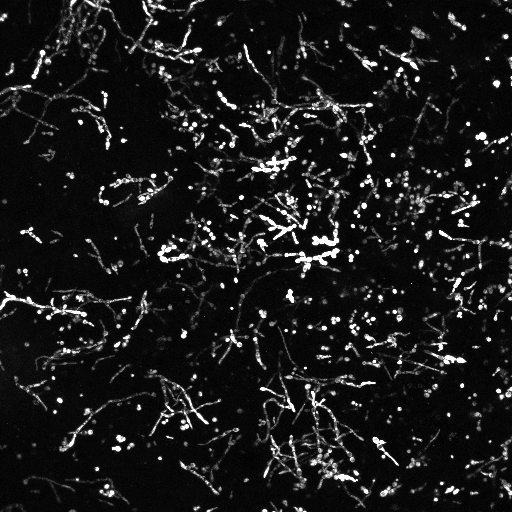

Supplement: Figure 2—figure supplement 1—source data 1. [file elife-85114-fig2-figsupp1-data1.zip › Figure 2-supplement 1-source data 1/P57055_Max Stack.tif]

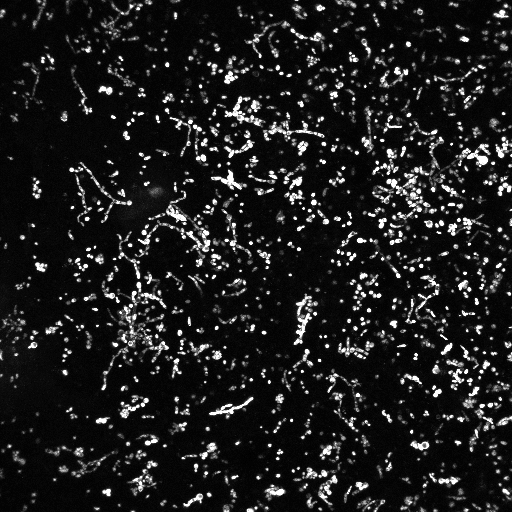

Supplement: Figure 2—figure supplement 1—source data 1. [file elife-85114-fig2-figsupp1-data1.zip › Figure 2-supplement 1-source data 1/P57055-ume6_Max Stack.tif]

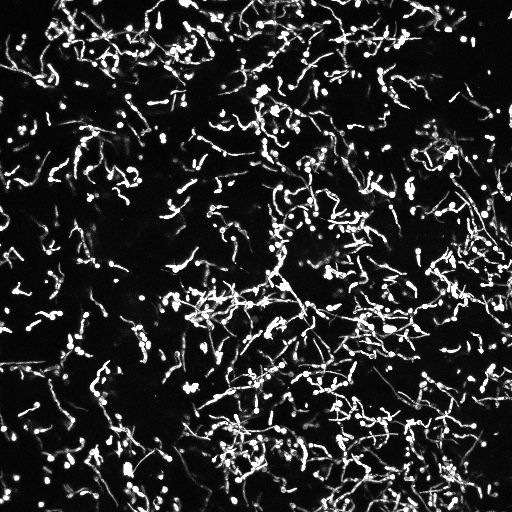

Supplement: Figure 2—figure supplement 1—source data 1. [file elife-85114-fig2-figsupp1-data1.zip › Figure 2-supplement 1-source data 1/P76067_Max Stack.tif]

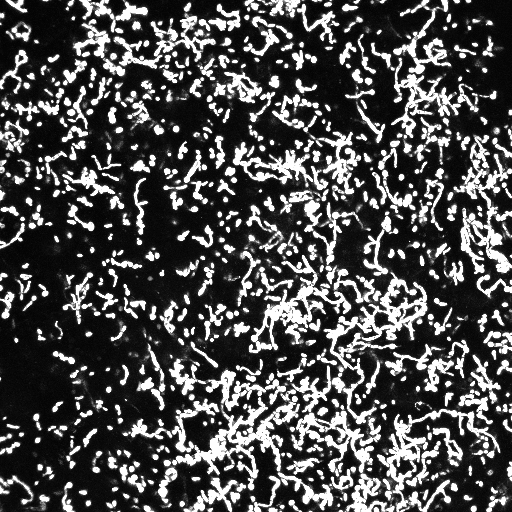

Supplement: Figure 2—figure supplement 1—source data 1. [file elife-85114-fig2-figsupp1-data1.zip › Figure 2-supplement 1-source data 1/P76067-ume6_Max Stack.tif]

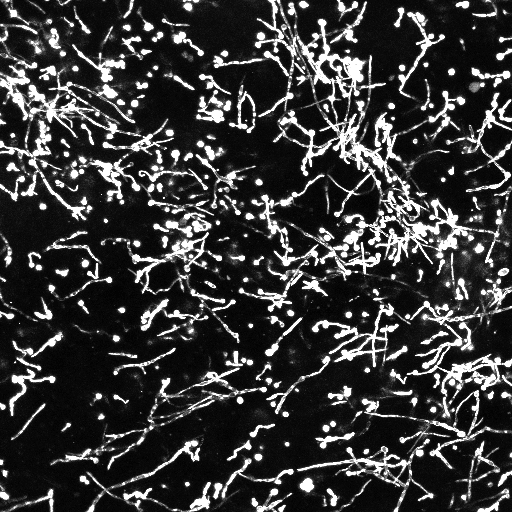

Supplement: Figure 2—figure supplement 1—source data 1. [file elife-85114-fig2-figsupp1-data1.zip › Figure 2-supplement 1-source data 1/P87_Max Stack.tif]

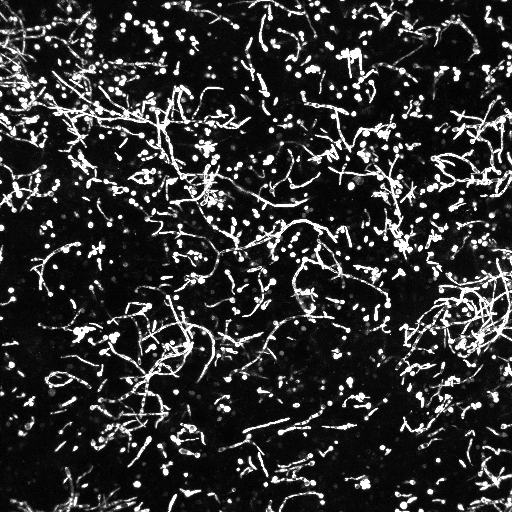

Supplement: Figure 2—figure supplement 1—source data 1. [file elife-85114-fig2-figsupp1-data1.zip › Figure 2-supplement 1-source data 1/P87-ume6_Max Stack.tif]

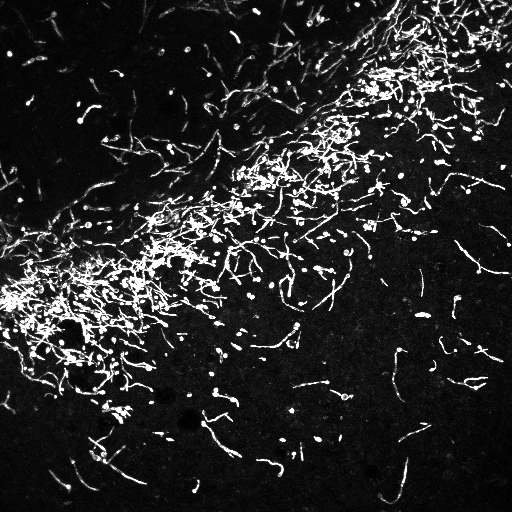

Supplement: Figure 2—figure supplement 1—source data 1. [file elife-85114-fig2-figsupp1-data1.zip › Figure 2-supplement 1-source data 1/SC5314_Max Stack.tif]

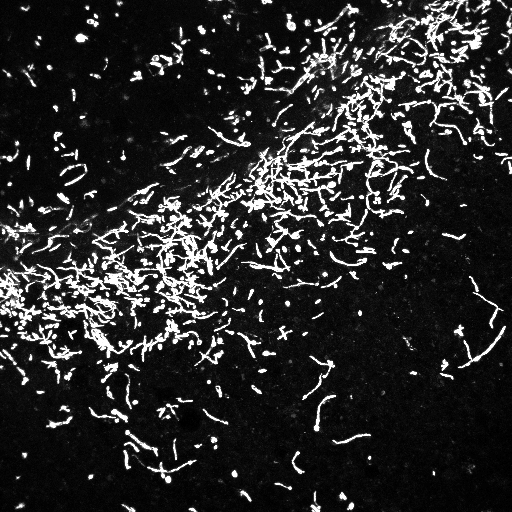

Supplement: Figure 2—figure supplement 1—source data 1. [file elife-85114-fig2-figsupp1-data1.zip › Figure 2-supplement 1-source data 1/SC5314-ume6_Max Stack.tif]

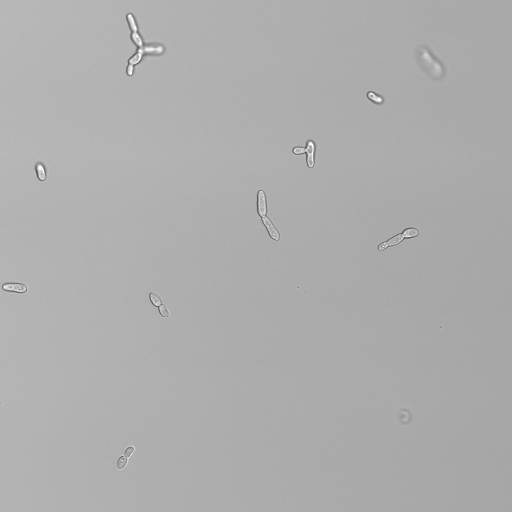

Supplement: Figure 8—source data 1. — Images for comparison analysis of efg1∆∆ nrg1∆∆ and efg1∆∆ tup1∆∆ filamentation. [file elife-85114-fig8-data1.zip › Figure 8. A-source data 1/efg1-tup1_RPMI+10%Serum_4hr.tif]
