# Supplementary material for: Intravital imaging-based genetic screen reveals the transcriptional network governing Candida albicans filamentation during mammalian infection
Source: eLife. 2023 Feb 27;12:e85114. doi: 10.7554/eLife.85114 (PMC9995110; doi:10.7554/eLife.85114)
Supplement: Supplementary file 8. [file elife-85114-supp8.docx]

**Supplementary File 9: Strain Table**

| **Strain** | **Genotype** |  |
| --- | --- | --- |
| SC5314 | Wild type clinical isolate | [1] |
| SN250 | *arg4*Δ/*arg42*Δ *URA3*/*ura3*Δ::*imm 434* *IRO1/iro1Δ*::*imm 434* | [2] |
| SN152 | \| CAI4 with *iro1::IRO1/iro1*::*λ*imm434 *his*1::*hisG*/*his1*::*hisG leu2∆/leu2∆ arg4∆/arg4∆* \| \| --- \| | [2] |
| *efg1Δ/Δ* | As SN152 with *efg1Δ::HIS1/ efg1Δ::LEU3* | [2] |
| *ume6Δ/UME6* | As SN152 with *ume6Δ :: HIS1/* *UME6* | This Study |
| *ume6Δ/Δ* | As SN152 with *ume6Δ :: HIS1/* *ume6Δ ::ARG4* | This Study |
| *brg1Δ/Δ* | As SN152 with *brg1Δ*::*ARG4*/*brg1Δ::ARG4* | This Study |
| \| *rob1Δ/Δ* \| \| --- \| | As SN152 with *rob1Δ::HIS1/rob1Δ::HIS1* | This Study |
| *brg1Δ/Δ nrg1Δ/Δ* | As *brg1Δ/Δ* with *nrg1Δ::HIS1/nrg1Δ/Δ*::*HIS1* | This Study |
| *rob1Δ/Δ nrg1Δ/Δ* | As *rob1Δ/Δ* with *nrg1Δ::ARG4/nrg1Δ/Δ*::*ARG4* | This Study |
| *efg1Δ/Δ nrg1Δ/Δ* | As *efg11Δ/Δ* with *nrg1Δ::ARG4/nrg1Δ/Δ*::*ARG4* | This Study |
|  |  |  |

1. Gillum, A.M., E.Y. Tsay, and D.R. Kirsch, *Isolation of the Candida albicans gene for orotidine-5'-phosphate decarboxylase by complementation of S. cerevisiae ura3 and E. coli pyrF mutations.* Mol Gen Genet, 1984. **198**(2): p. 179-82.

2. Noble, S.M. and A.D. Johnson, *Strains and strategies for large-scale gene deletion studies of the diploid human fungal pathogen Candida albicans.* Eukaryot Cell, 2005. **4**(2): p. 298-309.
